# Targeting alarmin release reverses Sjogren's syndrome phenotype by revitalizing Ca^2+^ signalling

**DOI:** 10.1002/ctm2.1228

**Published:** 2023-04-03

**Authors:** Yuyang Sun, Viviane Nascimento Da Conceicao, Arun Chauhan, Pramod Sukumaran, Pooja Chauhan, Julian L. Ambrus, Arjan Vissink, Frans G. M. Kroese, Madesh Muniswamy, Bibhuti B. Mishra, Brij B. Singh

**Affiliations:** ^1^ Department of Periodontics School of Dentistry University of Texas Health Science Center San Antonio San Antonio Texas USA; ^2^ Department of Developmental Dentistry School of Dentistry University of Texas Health Science Center San Antonio San Antonio Texas USA; ^3^ Department of Biomedical Sciences School of Medicine and Health Sciences University of North Dakota Grand Forks North Dakota USA; ^4^ Division of Allergy, Immunology, and Rheumatology Department of Medicine School of Medicine and Biomedical Sciences State University of New York Buffalo New York USA; ^5^ Department of Oral and Maxillofacial Surgery University of Groningen and University Medical Center Groningen Groningen The Netherlands; ^6^ Department of Rheumatology and Clinical Immunology University of Groningen and University Medical Center Groningen Groningen The Netherlands; ^7^ Department of Medicine University of Texas Health Science Center San Antonio San Antonio Texas USA

**Keywords:** alarmins, Ca^2+^ signalling, ER stress, immune cell activation, primary Sjogren's syndrome, salivary gland dysfunction

## Abstract

**Background:**

Primary Sjogren's syndrome (pSS) is a systemic autoimmune disease that is embodied by the loss of salivary gland function and immune cell infiltration, but the mechanism(s) are still unknown. The aim of this study was to understand the mechanisms and identify key factors that leads to the development and progression of pSS.

**Methods:**

Immunohistochemistry staining, FACS analysis and cytokine levels were used to detect immune cells infiltration and activation in salivary glands. RNA sequencing was performed to identify the molecular mechanisms involved in the development of pSS. The function assays include in vivo saliva collection along with calcium imaging and electrophysiology on isolated salivary gland cells in mice models of pSS. Western blotting, real‐time PCR, alarmin release, and immunohistochemistry was performed to identify the channels involved in salivary function in pSS.

**Results:**

We provide evidence that loss of Ca^2+^ signaling precedes a decrease in saliva secretion and/or immune cell infiltration in IL14α, a mouse model for pSS. We also showed that Ca^2+^ homeostasis was mediated by transient receptor potential canonical‐1 (TRPC1) channels and inhibition of TRPC1, resulting in the loss of salivary acinar cells, which promoted alarmin release essential for immune cell infiltration/release of pro‐inflammatory cytokines. In addition, both IL14α and samples from human pSS patients showed a decrease in TRPC1 expression and increased acinar cell death. Finally, paquinimod treatment in IL14α restored Ca^2+^ homeostasis that inhibited alarmin release thereby reverting the pSS phenotype.

**Conclusions:**

These results indicate that loss of Ca^2+^ signaling is one of the initial factors, which induces loss of salivary gland function along with immune infiltration that exaggerates pSS. Importantly, restoration of Ca^2+^ signaling upon paquinimod treatment reversed the pSS phenotype thereby inhibiting the progressive development of pSS.

## Graphical abstract

1. Loss of ^Ca2+^ signaling is critical for the development of pSS pathogenesis.
2. Induction of ER stress that releases alarmins that promotes immune cell migration in pSS.
3. Paquinimod treatment restores ^Ca2+^ signaling and prevent alarmin release that reverts the pSS pathogenesis in pre‐clinical mouse models.

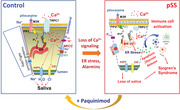

## 1 INTRODUCTION

Primary Sjogren's syndrome (pSS) is characterized as a chronic autoimmune disease that specifically affects women leading to dysfunctions of the exocrine gland.^1^, ^2^, ^3^, ^4^ Although lymphocytic infiltration is common in salivary glands, extra glandular signs are also observed for pSS patients that include enhanced lymphoproliferation with an increased risk for non‐Hodgkin's B‐cell lymphoma.^5^, ^6^ pSS patients also develop other complications that include fatigue, rash, arthritis, interstitial lung disease, nephritis and neuropathy.^7^, ^8^ In addition, pSS patients also showed hypergammaglobulinemia and autoantibodies that are directed towards SSA (Ro), SSB (La), muscarinic receptors, and a variety of other salivary gland antigens have been also reported.^9^, ^10^ Although the loss of stimulated and unstimulated saliva flow and abnormal immune regulation are present in pSS patients and animal models of pSS, the mechanism that initiates the disease is not well understood.^11^, ^12^ Moreover, numerous clinical trials using targeted immunomodulatory therapies have shown limited or no benefits for pSS patients.^13^, ^14^, ^15^ In contrast, the addition of pilocarpine, which induces Ca^2+^ signalling, has been able to improve the symptoms of dry mouth and dry eyes in these patients. Together, these results suggest that pSS is perhaps not initiated due to the overactivation of the immune cells, and other mechanisms may be critical in the development/progression of the disease. In addition, although saliva is severely reduced in pSS patients, the number of acinar cells that produce saliva remains relatively constant and does not correlate with disease severity. Moreover, the gross morphology of salivary glands appears normal in pSS samples suggesting that perhaps those remaining acinar cells remain nonfunctional,^11^ again suggesting that factors modulating salivary gland function might be more critical.

Saliva is essential for oral health, and salivary fluid is physiologically important for modulating functions, such as speech modulation and efficient mastication.^16^ In addition, saliva is critical for buffering the oral and stomach pH, maintaining the oral microbiome, along with maintaining tooth mineralization.^17^, ^18^ Similarly, it also facilitates wound healing, along with neutralizing harmful dietary components, lubricating and hydrating oral mucosal surfaces.^17^, ^18^ Importantly, Ca^2+^ signalling machinery is shown to be critical for saliva secretion in higher mammals. Release of the neurotransmitter “Acetylcholine” from parasympathetic nerves activates the muscarinic M3 receptors to generate inositol 3 triphosphate (IP_3_) that releases endoplasmic reticulum (ER) Ca^2+^, upon binding to the IP_3_Rs. Depletion of ER Ca^2+^ allows STIM1 to multimerize and interact with Ca^2+^ entry channels to initiate Ca^2+^ entry (termed store‐operated Ca^2+^ entry [SOCE]).^19^, ^20^ This elevated intracellular Ca^2+^ further modulates other ion‐transporting proteins to induce saliva secretion.^21^ Members of the transient receptor potential (TRP) superfamily and Orai1 have been shown to function as Ca^2+^ channels thereby regulating Ca^2+^ entry upon store depletion.^19^, ^20^, ^22^ The TRP canonical‐1 (TRPC1) channel is present in salivary tissues and has been suggested as a component of SOCE.^23^ Our previous studies have identified TRPC1 as an important regulator for saliva secretion.^24^ TRPC1^−/−^ mice exhibit decreased saliva, which was due to the decrease in Ca^2+^ entry.^25^ Importantly, IL‐17, which contributes to salivary gland dysfunction, has also been shown to decrease TRPC1 function^26^; however, if TRPC1 can lead to pSS remains largely unknown.

Besides Ca^2+^ signalling, ER is also needed for protein synthesis and folding. Protein quality control is essential for the elimination of the unfolded or misfolded protein, a condition known as the unfolded protein response (UPR), which leads to ER stress.^27^ Importantly, loss of ER function, which leads to ER stress, has been identified to play a vital role in the pathogenesis of diseases, such as neuronal function/neurodegenerative disorders, obesity, diabetes, cancer and inflammatory diseases. Importantly, autoimmune diseases have increased inflammation, along with an increase in ER stress conditions. Similarly, damage‐associated molecular patterns (DAMPs/alarmins) that activate the immune responses could induce inflammation and promote cell death^28^; however, if similar mechanism(s) are present in pSS is not clear. The activation of the UPR pathway further stimulates several transcription factors such as NFκB, XBP1s, AP‐1ATF6 and the cAMP response element‐binding protein‐H, which modulates the pro‐inflammatory cytokines/chemokines and induces the activation of the inflammatory response. Furthermore, the ER stress is also important for the activation of CD4^+^ and CD8^+^ T cells upon antigen recognition,^29^, ^30^ which contributes to pSS.

Sjogren's syndrome patients have increased expression of IL‐14α, and mice that constitutively overexpress IL‐14α developed features that are similar to pSS, such as hypergammaglobulinemia, autoantibodies, lymphocyte infiltration in salivary glands and large B‐cell lymphoma.^31^ Although IL14 is not implicated in ER stress, the IL14α Tg mice showed features of pSS in a similar and thus is a good model for pSS.^32^, ^33^ Thus, to understand the mechanisms and identify factors that lead to the development and progression of pSS, this study was performed. The data presented here indicate that IL14α Tg mice developed pSS‐like histological appearance in salivary glands over time; however, both decreases in saliva secretion and salivary gland cell death were observed before immune infiltration. Interestingly, loss of Ca^2+^ entry led to a decrease in saliva secretion that induces immune cell infiltration. TRPC1 expression in salivary gland cells was decreased in both the pSS and IL14α Tg mouse model which could account for the loss of Ca^2+^ signalling and decrease in saliva secretion. Moreover, TRPC1^−/−^ mice showed increased immune infiltration and abnormal immune cell presence in salivary glands. Finally, mechanistically we show that reduction in TRPC1‐mediated Ca^2+^ entry leads to the loss of salivary gland cells, followed by the release of alarmins that promoted immune cells infiltration into salivary glands. Moreover, blocking alarmins through paquinimod^34^, ^35^, ^36^ not only increased Ca^2+^ signalling but also restored saliva secretion, showed decreased immune activation and prevented the development of the pSS phenotype in IL14α Tg mouse model.

## 2 RESULTS

### 2.1 Glandular loss precipitates lymphocytic infiltration in salivary glands

**FIGURE 1:**
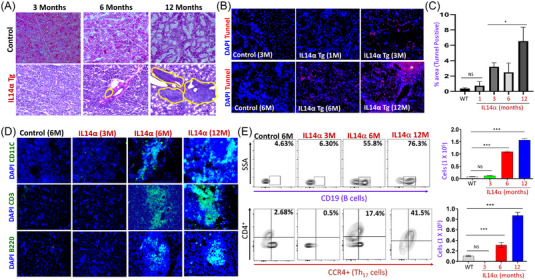
Loss of salivary glands precedes immune cell migration: Part (A) shows hematoxylin and eosin (H&E) staining in salivary glands from control and IL14α Tg mice among various age‐groups (images shown are representative of 3–4 animals from each age‐group). The presence of immune cells is marked by a yellow border. (B) Representative confocal images showing apoptosis (using tunnel staining) in salivary glands from control and IL14α Tg mice. Age‐groups of animals tested are indicted in the figure. “*” Indicates cell death in acinar cells. Part (C) shows quantification of the tunnel^+^ cells in various conditions from 3 to 5 individual samples, *p ≤ .001 (Student's *t* test). (D) Confocal images showing the presence of CD11c (marker for dendritic cells), CD3 (marker for T cells) and B220 (marker for B cells) in salivary glands from control and IL14α Tg mice. Age‐groups used are indicted in the figure, and the images shown are representation of three separate experiments. (E) Lymphocytes were isolated from salivary glands of IL14α mice (1, 6 and 12 months old) and control littermates. Cells were stained with anti‐CD19 (PerCP‐Cy5.5), anti‐CD4 (phycoerythrin [PE]) and anti‐CCR4 (allophycocyanin [APC]) using multi‐colour flow cytometry. Gates were used to determine the percentage of infiltrating immune cells in the salivary gland tissues of the mice, CD19^+^ high (B‐cell marker) as indicated and the combined CD4^+^ high (T cell marker) and CCR4^+^ high (TH17 cell marker) for each mice group and control. Bar graphs represent the number of total number cells marked positively for the specific markers. Data shown are representative of three‐independent experiments with similar results. Bar graphs depict average ± SD for relative values, ***p ≤ .001, NS = non‐significant (Student's *t* test).

The most common feature observed in pSS patients is the loss of saliva and lymphocytic infiltration in salivary glands. Thus, we initially evaluated the presence of immune cells and observed stimulated saliva in age‐matched control (non‐SS) or pSS patients (Figure S1A). Importantly, a significant increase in the lymphocytic inflammation in salivary tissues, which was determined by focus scoring (aggregate of at least 50 lymphocytes/4 mm^2^), was observed in pSS patients when compared with age‐matched controls (Figure S1B,C). Similarly, a significant decrease in stimulated saliva secretion was also observed in pSS patients as compared with control patients (Figure S1B,C). Although patient data show that both decreased saliva secretion and an increase in glandular infiltration of immune cells are observed in pSS, the timeline/sequence for events, and if these events are linked, is not known. Thus, we used age/sex/genetic‐matched IL14α transgenic (IL14α Tg) mice that exhibit similar characteristics as observed in pSS and so could be used as a model for pSS. Like control mice, salivary glands obtained from 3 months of age of IL14α Tg mice showed normal salivary gland morphology, and no evidence of lymphocytic infiltration was observed (Figure 1A). Similar results were also obtained in 1‐month‐old‐L14α Tg mice (data not shown). However, 6‐month‐old IL14α Tg mice showed a diffuse immune cell infiltration, especially near the blood vessels (perivascular areas), which was further increased in 12‐month‐old IL14α Tg mice (Figure 1A). Importantly, 12‐month IL14α Tg mice displayed a further increased in immune cells presence with larger and multiple foci formation throughout the salivary tissue (Figure 1A). Overall, the hematoxylin and eosin (H&E) staining showed an age‐dependent increase of infiltrating immune cells in salivary glands of IL14α Tg mice.

In comparison, IL14α Tg salivary tissues also exhibited similar morphology with minimal tunnel+ staining at 1 month of age (Figure 1B). However, IL14α Tg mice at 3 months of age displayed an increased tunnel+ staining in salivary glands, which was further significantly increased at 6 and 12 months of age (Figure 1B,C). Importantly, cell death observed in the salivary glands of 6‐month‐old IL14α Tg mice was more widespread, which was different from lymphocytic infiltration that showed a much‐limited presence that too mainly in the perivascular areas. To establish the kinetics involved in the accumulation of various immune cell types in IL14α Tg mice, we used immunofluorescence microscopy to determine the presence and extent of accumulation of immune cells in various age‐groups (Figure 1C and Figure S1D). Likewise, in IL14α Tg mice salivary gland tissue, T cells (positive for CD3^+^), B cells (positive for B220^+^) and monocytes/dendritic cells (CD11c^+^ cells) were detected only after 6 months, and both 1 (data not shown) or 3‐month‐old mice did not show any increase in immune cell infiltration. As expected, the control mice salivary gland (6 months) showed the absence of CD11c^+^ cells, and a complete lack of B and T cells were observed at all the time points tested. The gating strategy used to analyse individual markers related to activation status with the identification of immune cells is shown in Figure S1E. Flow cytometry further showed a significant increase in the number of CD19 positive cells (for B cells) and Th_17_ positive T cells along with an increase in the number of monocytes (CD11b^+^) in 6–12‐month‐old IL14α Tg mice, whereas minimal immune cells were observed in control (6 months) or 1‐month‐old IL14α Tg mice (except for monocytes, which were higher in 3‐month‐old IL14α Tg mice (Figure 1D and Figure S1F)). These results are consistent with previous reports showing immune infiltration in IL14α Tg mice at a later age Refs. [32, 33]. Taken together, these data demonstrate that perhaps early pathology in the salivary gland likely contributes to a significant increase in leukocyte recruitment and infiltration.

### 2.2 The loss of salvia secretion is observed within 6 months in IL14α Tg mice

**FIGURE 2:**
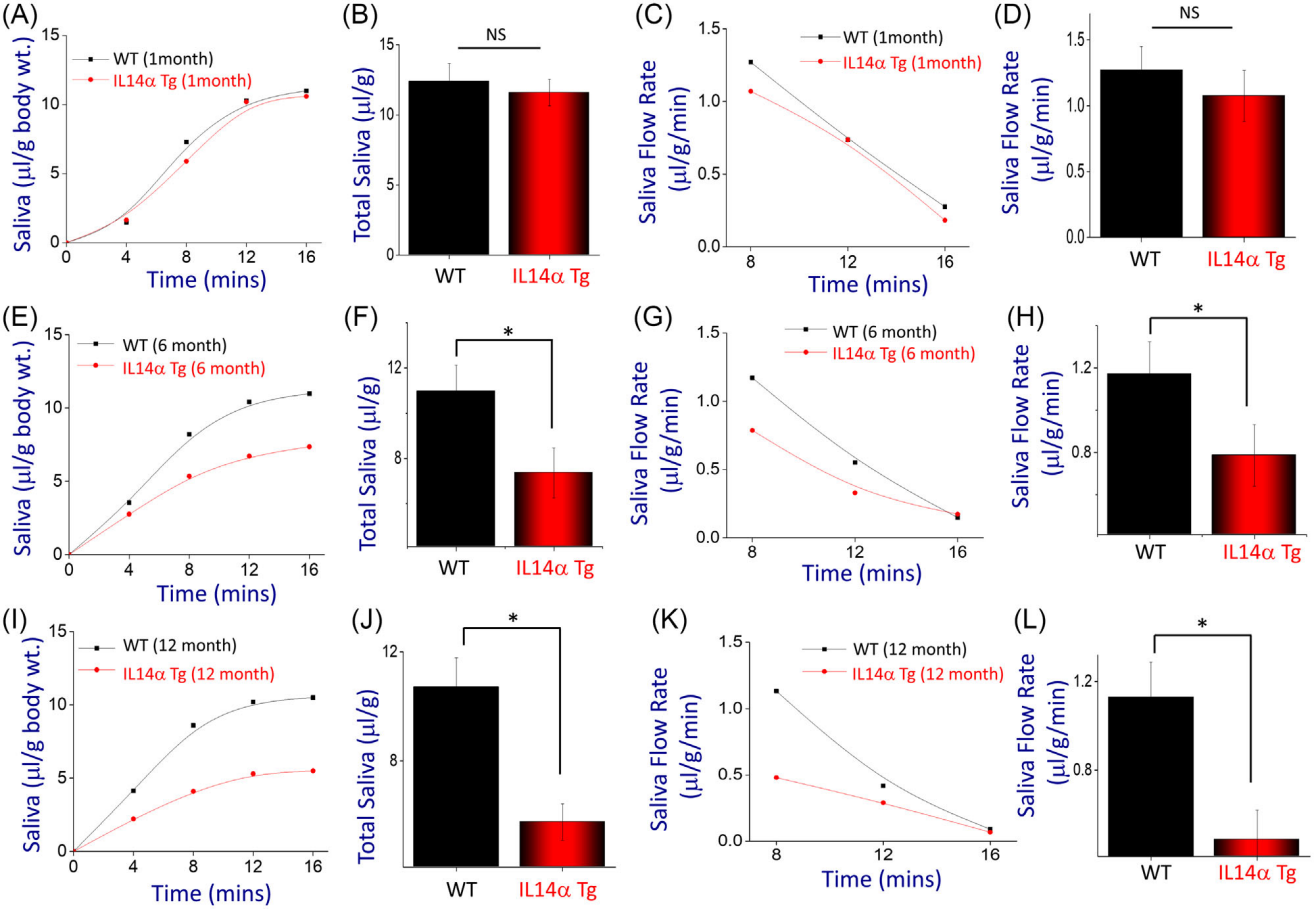
Age‐dependent decrease in salivary gland function is observed in IL14α Tg mice: (A) saliva secretion was induced by pilocarpine in 1‐month‐old control (wild‐type [WT]) and IL14α Tg mice and plotted. The data presented are representative of 6–8 animals in each group. Saliva was collected every 4 min, and total saliva secreted is plotted as line graph. (B) Quantification of total saliva (6–8 mice) secreted in 1‐month‐old control (WT) and IL14α Tg mice is shown as bar graph. Error bars represent means ± SE. NS indicates no significant difference between the two groups. (C) Saliva flow rate in 1‐month‐old control (WT) and IL14α Tg mice is shown as line graph, which showed a time‐dependent decrease in saliva secretion. (D) Quantification of saliva flow rate (8–10 mice) in 1‐month‐old control (WT) and IL14α Tg mice is shown as bar graph. Error bars represent means ± SE. NS indicates no significant difference between the two groups. (E) Saliva secretion in 6‐month‐old control (WT) and IL14α Tg mice (6–10 mice) and plotted. (F) Quantification of total saliva secreted (6–8 mice for each group) in 6‐month‐old control (WT) and IL14α Tg mice is shown as bar graph. Error bars represent means ± SE. *p ≤ .001 (Student's *t* test). (G) Saliva flow rate in 6‐month‐old control (WT) and IL14α Tg mice is shown. Quantification of saliva flow rate (6–8 mice) in 6‐month‐old control (WT) and IL14α Tg mice is shown as bar graph (H). Error bars represent means ± SE. *p ≤ .001 (Student's *t* test or analysis of variance [ANOVA]). (I) Secretion of saliva and its quantification is shown as bar graph in (J) in 12‐month‐old control (WT) and IL14α Tg mice (6 mice each). Error bars represent mean ± SE. *p ≤ .001 (Student's *t* test or ANOVA). (K) Saliva flow rate in 12‐month‐old control (WT) and IL14α Tg mice is shown. Quantification of saliva flow rate (8 mice) in 12‐month‐old control (WT) and IL14α Tg mice is shown as bar graph (L). Error bars represent mean ± SE. *p ≤ .001.

To determine when salivary gland function is inhibited, we next evaluated saliva secretion in control and IL14α Tg mice of different age‐groups. Importantly, stimulation with pilocarpine showed no significant difference in the total saliva secretion in 1‐month‐old IL14α Tg mice when compared with age‐matched control mice (Figure 2A,B). Similarly, the rate of saliva secretion (for the first 12 min) was also not significantly decreased in 1‐month‐old IL14α Tg mice when compared with age‐matched controls (Figure 2C,D). Similar results were also obtained from 3‐month‐old IL14α Tg mice, where no significant decrease in saliva secretion was observed (data not shown). In contrast, 6‐month‐old IL14α Tg mice showed a significant decrease in saliva secretion when compared to age‐matched controls (Figure 2E,F). Interestingly, 6‐month‐old wild‐type (WT) mice displayed a typically high rate of saliva secretion immediately after stimulation that decreased with time, whereas the IL14α Tg mice showed consistently low saliva secretion (Figure 2G,H). These results further provide evidence that perhaps it is not the lymphocytic infiltrations per se (as the number of immune cells was low at 6 months as shown in Figure 1) that leads to a decrease in glandular function in IL14α Tg mice. Importantly, 12‐month‐old IL14α Tg mice further showed a decrease in both the total saliva secreted and the rate of saliva secretion as compared with age‐matched controls (Figure 2I–L). Overall, data presented above suggest that the loss of salivary gland function may be an early event that could lead to immune cell infiltration as observed in pSS.

**FIGURE 3:**
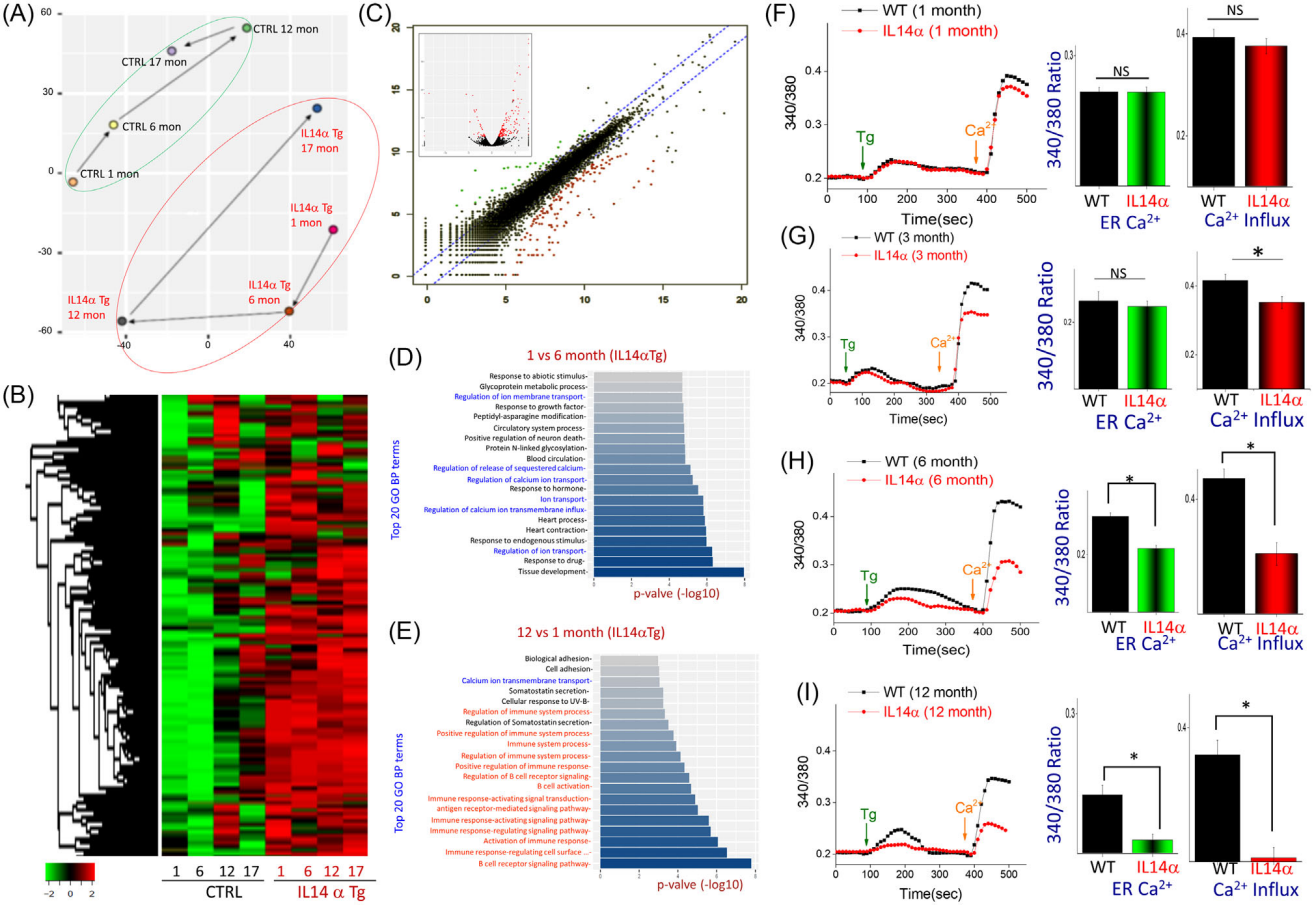
Age‐induced gradual loss of Ca^2+^ signalling in IL14α Tg mice: (A) principal component analysis (PCA) plot of normalized FPKM of RNA‐seq from duplicate samples of various age‐groups in control and IL14α Tg mice. (B) Heat map and clustering based on hierarchy showing differential expression of genes in control and IL14α Tg mice (at age 1, 6, 12 and 17 months). (C) Differential expression of genes in the salivary glands between control (6 months) and IL14α Tg mice (6 months). Volcano plot indicating up‐regulated and down‐regulated genes in control and IL14α Tg mice is shown as inset. Top gene ontology (GO) terms showing top 20 significant biological process and their molecular functions, in various pathways, are labelled. The data used are the −log10 *p* values from the differential expression (decreased) list of 1 versus 6 months (D) and increased in 12 versus 1 month (E) old IL14α Tg mice. (F) Representative plots of Ca^2+^ imaging performed on Fura‐2AM loaded primary acinar cells isolated from submandibular glands of 1‐month‐old control (wild‐type [WT]) and IL14α Tg mice (*n* = 3 mice each). Cells were stimulated by Tg (1 µM) followed by the addition of 1 mM external Ca^2+^. Bar graphs (shown in right) indicate quantification of the endoplasmic reticulum (ER) Ca^2+^ release and Ca^2+^ entry in data from more than 90–120 cells/areas in each condition and is plotted as mean ± SEM. NS indicates no significant difference between the two groups. (G) Individual traces showing changes in Ca^2+^ imaging in primary acinar cells isolated from submandibular glands of 3‐month‐old control (WT) and IL14α Tg mice (*n* = 3 mice each). Quantification of the ER Ca^2+^ release and Ca^2+^ entry from more than 120 cells in each condition is plotted as mean ± SEM in the bar graphs. *p < .05 indicates values that are significantly different from WT acinar cells (Student's *t* test). (H) Ca^2+^ imaging traces from primary acinar cells isolated from 6‐month‐old control (WT) and IL14α Tg mice. Bar graphs indicate quantification of the ER Ca^2+^ release and Ca^2+^ entry in data from more than 100 cells in each condition and is plotted as mean ± SEM. *p < .05 indicates values that are significantly different from WT acinar cells (Student's *t* test). (I) Ca^2+^ imaging trace in primary acinar cells isolated from submandibular glands of 12‐month‐old control (WT) and IL14α Tg mice. Quantification of the ER Ca^2+^ release and Ca^2+^ entry from more than 90 cells in each condition is plotted as mean ± SEM in the bar graphs. *p < .05 indicates values that are significantly different from WT acinar cells (Student's *t* test).

To further understand the reasons as why saliva secretion is inhibited as the mice aged, we used a genomic approach, and total RNA was isolated from 1‐, 6‐, 12‐, and 17‐month‐old control and IL14α Tg mice. Importantly, the dynamism of RNA clustering was tight suggesting that cellular transcriptome changes could be possible in IL14α Tg mice (Figure 3A), which could account for the development of the phenotype. Moreover, the heat map showed a significant change in the expression of a subset of genes that were either gradually decreased or increased in IL14α Tg mice (upon aging), when compared with control mice (Figure 3B). Scatter plot further showed that expression of a subset of genes was altered in 6‐month‐old IL14α Tg mice (Figure 3C). Importantly, GO enrichment analysis between 1‐ and 6‐month‐old IL14α Tg mice showed a significant loss in the RNA levels of key ion channels genes that were involved in ion signalling, including Ca^2+^ signalling (Figure 3D), suggesting that they are affected within 6 months of age in IL14α Tg mice. However, a comparison of genes that were increased in 1‐ versus 12‐month‐old IL14α Tg mice showed a significant increase in genes that are involved in immune‐related pathways (Figure 3E), which could be due to the increase in the presence of immune cells. Interestingly, a comparison between WT and IL14α Tg mice showed that most of the genes involved the immune pathways (BCR and TCR) were increased, whereas all major signalling pathways were decreased (Figure S2A,B). A table with the top 100 genes up‐regulated and down‐regulated is shown in Figure S3. RNA‐seq analysis also revealed ∼1000 differentially expressed genes between WT and IL IL14α Tg mice, which were either up‐regulated or down‐regulated (>twofold‐change) (Figure S2C,D). Interestingly, neurotransmitter‐mediated fluid secretion in salivary gland is critically dependent on Ca^2+^ entry,^21^, ^37^ suggesting that loss of Ca^2+^ signalling genes might contribute to the pathology.

### 2.3 Decreased Ca^2+^ entry leads to the decrease in saliva secretion

To further establish if the loss of saliva secretion observed in IL14α Tg mice is due to a decrease in Ca^2+^ levels, we next investigated intracellular Ca^2+^ levels ([Ca^2+^]*_i_*) in freshly isolated primary acinar cells. Thapsigargin (Tg, 2 µM), a sarcoendoplasmic reticulum Ca^2+^ transport ATPase (SERCA) blocker that depletes intracellular Ca^2+^ stores and activates Ca^2+^ entry, was used.^38^ Importantly, thapsigargin (Tg, first peak) induced increase in [Ca^2+^]*_i_* was similar in isolated SMG cells from 1‐month‐old control or IL14α Tg mice (Figure 3F). Subsequently, the addition of 1 mM external Ca^2+^, which initiates Ca^2+^ entry, was although slightly decreased but was not significantly different in cells isolated from 1‐month‐old IL14α Tg mice (Figure 3F). In contrast, acinar cells isolated from 3‐month‐old mice showed a reduction in Ca^2+^ entry, without any change in ER Ca^2+^ levels (Figure 3G). However, cells obtained from the submandibular glands of 6‐month‐old IL14α Tg mice showed a significant decrease in both the ER Ca^2+^ levels (peak upon Tg release in the absence of external Ca^2+^) and Ca^2+^ influx (Figure 3H). Moreover, acinar cells obtained from 12‐month‐old IL14α Tg mice further showed a significant decrease in both ER Ca^2+^ and Ca^2+^ influx, when compared with 12‐month‐old control mice (Figure 3I). Together, results presented thus far strongly suggest that decrease in intracellular Ca^2+^ levels may be an initial event that could lead to salivary gland dysfunction and contribute towards acinar cell loss, which is essential for immune infiltration and/or the pathology observed in pSS.

### 2.4 TRPC1‐like currents are decreased in aged IL14α Tg mice

**FIGURE 4:**
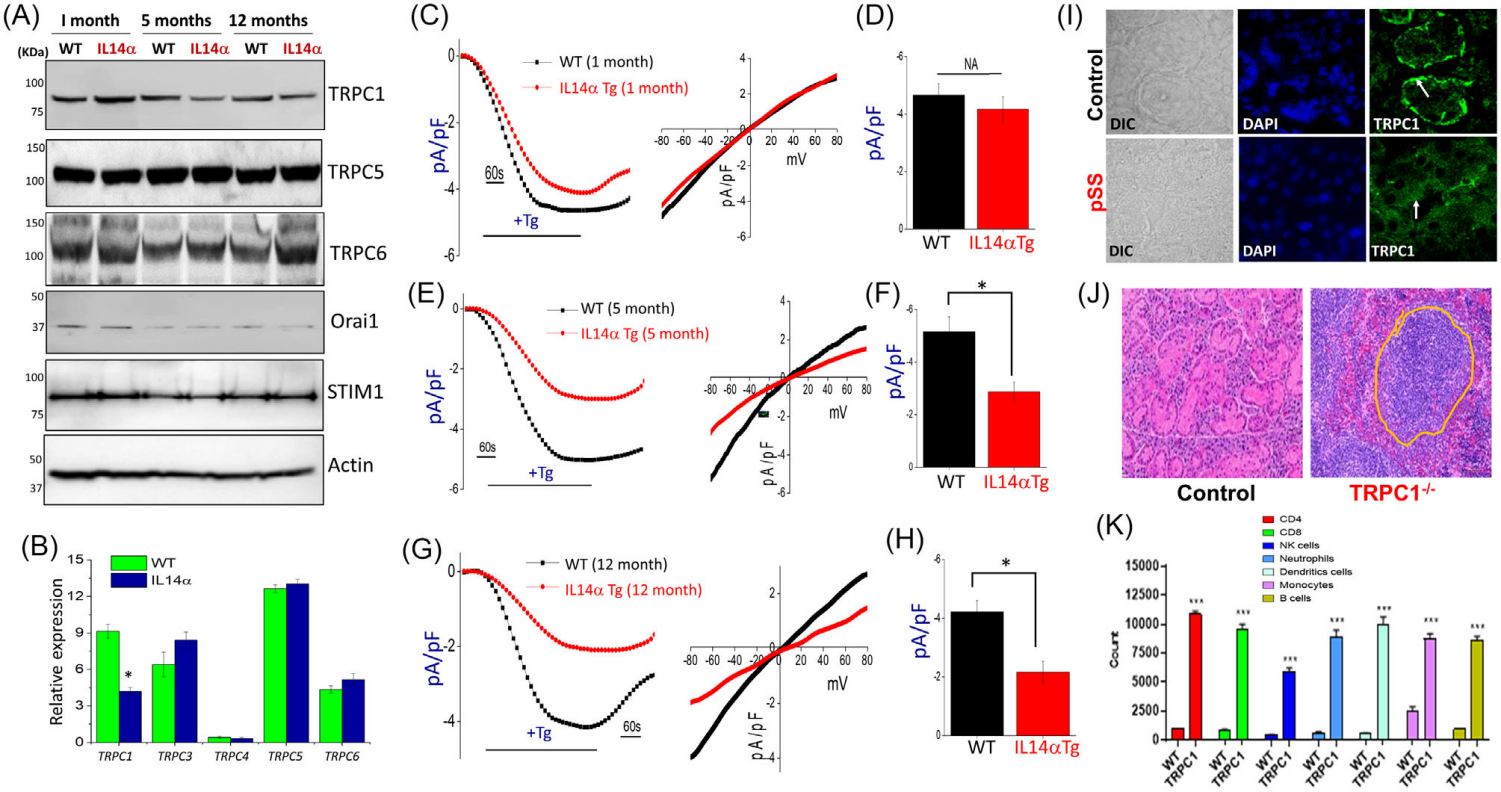
Transient receptor potential canonical‐1 (TRPC1)‐like currents are decreased over time in IL14α Tg mice: (A) Salivary gland (submandibular gland) lysates from control (wild‐type [WT]) and IL14α Tg mice samples (*n* = 4–6) from various age‐groups were subjected to SDS–PAGE and immunoblotted with respective antibodies as labelled. (B) Relative expression of TRPC1, TRPC3, TRPC4, TRPC5 and TRPC6 between WT mice and IL14α Tg mice (*n* = 3). mRNA levels of various TRPC genes in control and IL14α Tg mice (6 months). “*” Indicates significance *p* < .05. (C) Tg‐induced currents (at −80 mV) were evaluated in acinar cells isolated from 1‐month‐old control (WT) and IL14α Tg mice. Representative *I*/*V* curves developed from acinar cells isolated from 1‐month‐old control (WT) and IL14α Tg mice are potted. (D) The average current intensities (mean ± SEM) from 9 to 12 individual cells in each condition are shown as bar graph. NA indicates no significant difference between the two groups. (E) Tg‐induced currents (at −80 mV) were evaluated in acinar cells isolated from 5‐month‐old control (WT) and IL14α Tg mice. Representative *I*/*V* curves developed in these conditions are potted in part (F). pA/pF represents currents in picoamperes per picofarad. (F) The average current intensities (current/mean ± SEM) from 8–10 individual cells are shown as bar graph. * *p* < .05 indicates values that are significantly different from untreated WT acinar cells (Student's *t* test). (G) Tg‐induced currents (at −80 mV) were evaluated in acinar cells isolated from 12‐month‐old control (WT) and IL14α Tg mice. Representative *I*/*V* curves developed in these conditions are potted in part (G). (H) The average current intensities (mean ± SEM) from 8–15 individual cells are shown as bar graph. *p* < .05 indicates values that are significantly different from untreated WT acinar cells (Student's *t* test). Values are expressed as mean ± SE. In general, the “*”, “**” and “***” indicate *p* values less than .05, .01 and .001, respectively. (I) Confocal images showing the expression of TRPC1 in salivary glands from age‐matched (55 years old) control and primary Sjogren's syndrome (pSS) samples. Images shown are representation from three individual samples. Part (J) shows hematoxylin and eosin (H&E) staining in salivary glands from control and TRPC1^−/−^ (6‐month old, images are representation of three individual samples). (K) Bar graphs represent the total number of immune cells marked positively for the specific immune markers used in submandibular glands of control and TRPC1^−/−^. “***” Indicates significance *p* < .001. Data shown are representative of three‐independent experiments with similar results.

Given the importance of TRPC1‐mediated Ca^2+^ entry in salivary gland function, we next studied the expression of Ca^2+^ channel(s) and its modulators in these mouse models. As shown in Figure 4A, a decrease in TRPC1 expression was observed in the SMGs obtained from aged (5 or 12 months) mice, but not in 1‐month‐old IL14α Tg mice, when compared with control mice. In contrast, no change in the expression of other TRPC (TRPC5/6) or Orai1 channels was observed, though a gradual loss of Orai1 was observed in 5‐month‐old mice (Figure 4A and Figure S4A–C). In addition, the expression of the TRPC1 regulator, STIM1, which has been shown to functionally activate TRPC1/Orai1 channels, was also not altered among these different age‐groups. In addition, to the reduced protein levels, a decrease in TRPC1 RNA expression, but not for other TRPC isoforms, was observed in the submandibular gland isolated from 5‐month‐old IL14α Tg mice (Figure 4B). Importantly, our previous studies have proposed that TRPC1 is the Ca^2+^ entry, that is important for saliva secretion^23^, ^39^; however, recently Orai1 has also been shown to increase Ca^2+^ entry in various cells. Thus, to confirm the identity of the Ca^2+^ entry channel, which was decreased in IL14α Tg mice, whole‐cell currents were evaluated in submandibular gland acinar cells. Addition of thapsigargin (store depletion) immediately developed an inward current that reversed between 0 and +5 mV (Figure S4D). Importantly, the current has similar properties (pA/pF) that is observed with TRPC1 channels. Furthermore, the addition of a Ca^2+^ channel inhibitor, SKF‐96365,^27^ also prevented Ca^2+^ entry in salivary gland acinar cells (Figure S4D), suggesting that TRPC1 is perhaps the endogenous Ca^2+^ entry channels in salivary tissues of IL14α Tg mice. We next evaluated changes in Ca^2+^ currents in the different age‐groups of IL14α Tg mice. Importantly, no differences in thapsigargin‐induced Ca^2+^ currents were observed between acinar cells obtained from submandibular glands of 1‐month‐old IL14α Tg or control mice (Figure 4C,D). In contrast, similar to Ca^2+^ imaging data, thapsigargin‐induced Ca^2+^ currents were significantly reduced in acinar cells of 5‐month‐old IL14α Tg mice when compared with the control group (Figure 4E,F). However, the current properties did not change between the two groups as both the currents were linear and reversed at 0 and +5 mV. Similarly, primary acinar cells obtained from 12‐month‐old mice further showed a reduction in TRPC1‐like currents isolated from the submandibular glands of IL14α Tg mice, when compared with 12‐month‐old mice (Figure 4G,H). Overall, these results suggest that the loss of TRPC1‐mediated Ca^2+^ entry precedes loss of salivary gland function, thus, could be the main factor that contributes to pSS‐like pathology.

To further assess the importance of TRPC1 in pSS, we again used age‐matched control (non‐SICCA) and pSS samples. Importantly, TRPC1 was expressed in the basolateral region of acinar cells in control samples, which was not only decreased but also diffused staining of TRPC1 was observed in pSS patients (Figure 4I and Figure S4F). Importantly, like the Western blot, data that had no change in STIM1 expression or localization were observed (Figure S4F), suggesting that loss of TRPC1 could be the reason for decreased Ca^2+^ entry and salivary gland dysfunction as observed above. Next, we tested the consequence of the loss of TRPC1 function using human submandibular gland (HSG) cells. The addition of TRPC1 channel blocker SKF‐9365 decreased cell survival in a concentration‐dependent manner (Figure S4G). Similarly, expressions of the ER stress/cell death markers were increased in SKF‐96365‐treated cells (Figure S4H). To establish that the effects observed were specifically due to TRPC1, we inhibited TRPC1 expression. TRPC1‐silenced cells showed a loss of TRPC1 expression as compared with cells transfected with control siRNA (Figure S4H). Interestingly, an increase in ER stress marker, CHOP expression, was observed in TRPC1‐silenced cells (Figure S4H). Increased cell death was also observed in TRPC1‐silenced cells, which was further potentiated in cells that had both TRPC1siRNA and SKF treatment (Figure S4G). Importantly, salivary tissues of TRPC1^−/−^ mice showed immune infiltration, and an increase in all immune cells was observed in submandibular gland tissues (Figure 4J,K and Figure S4I). Together, these results suggest that loss of TRPC1 leads to a decrease in Ca^2+^ signalling, which potentiates the apoptotic loss of salivary cells.

### 2.5 Loss of salivary tissues induces alarmin release that activates immune cell infiltration

**FIGURE 5:**
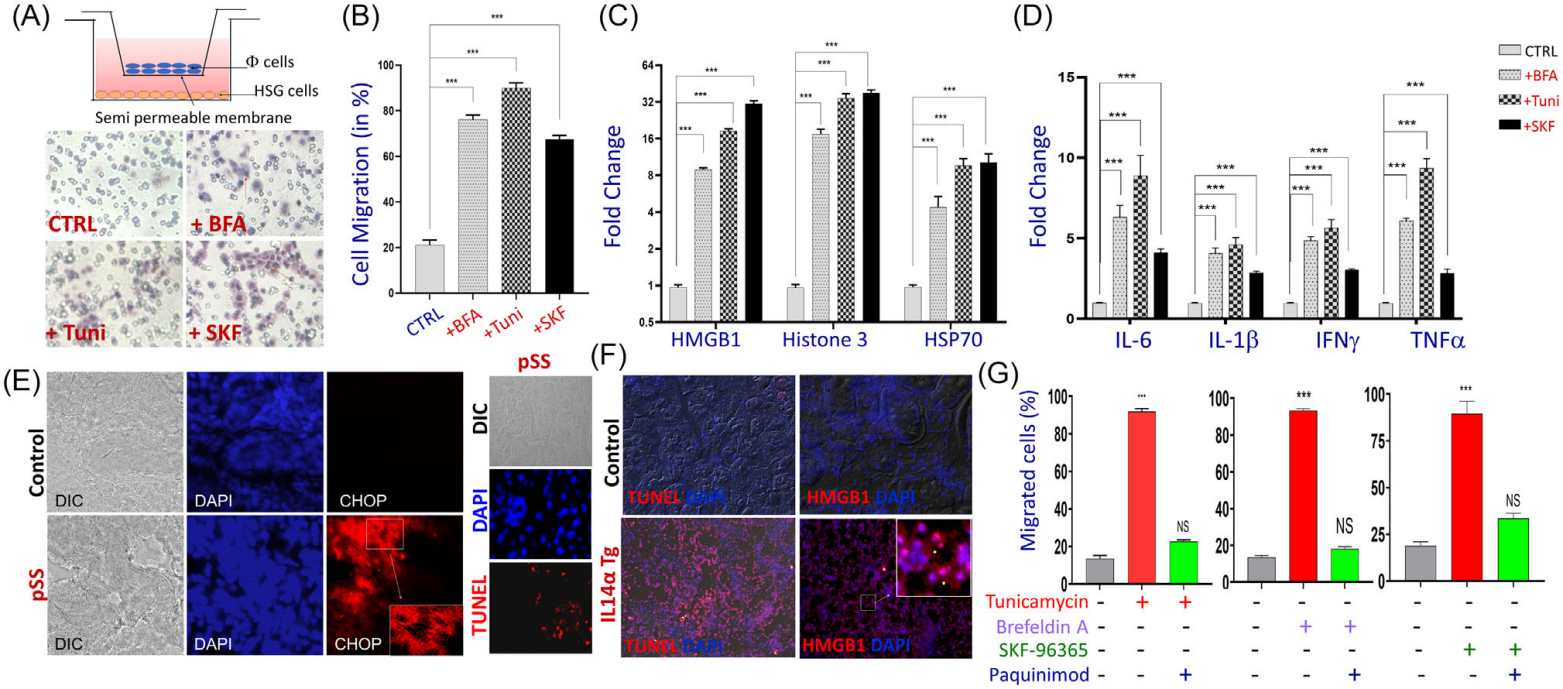
DAMPs released by human submandibular gland (HSG) cells after treatment with endoplasmic reticulum (ER) stress inducers promote immune cell migration and increase of inflammatory response. (A) Schematic representations of migration assay where 1 × 10^6^ HSG cells were plated in six wells plates in media containing tunicamycin (10 µM), brefeldin A (BFA) (10 µM), SKF96365 (10 µM) treated for 12 h. An insert is placed inside the wells with .5 × 10^6^ primary macrophages cells on the top, and then treatment is added in the lower part. Cells that migrated through the pores to the lower side of the insert membrane were fixed and counted at 40× magnification. (B) Data shown are representative of three‐independent experiments with similar results. Bar graphs depict average ± SD for positive cells, ***p ≤ .001 (Student's *t* test). (C) Damage‐associated molecular patterns (DAMPs) were analysed, by colorimetric analysis in supernatants of HSG cells after various treatments. Data shown are representative of three‐independent experiments with similar results. Bar graphs depict average ± SD for relative values, ***p ≤ .001 (Student's *t* test and/or analysis of variance [ANOVA]). (D) Pro‐inflammatory cytokines levels were analysed, by colorimetric analysis in supernatants (HSG cells and migrated macrophages) after treatment. Bar graphs (from three‐independent experiments) depict average ± SD for relative values, ***p ≤ .001 (ANOVA). (E) Confocal images showing the expression of CHOP (marker for ER stress) and tunel staining in salivary glands from age‐matched control and primary Sjogren's syndrome (pSS) samples. Images shown are representation of three individual experiments. (F) Confocal images showing HMGB1 expression in salivary glands from 6‐month‐old control and IL14α Tg mice. Images shown are representation of three individual experiments. (G) Bone marrow‐derived primary macrophages were also treated with supernatants of HSG cells treated with ER stress inducers (tunicamycin or BFA) or Ca^2+^ channel blocker (SKF‐96365) with and without paquinimod for 12 h, and then the media was used as chemoattracts for migration stimulation. Immune cell migration was measured in response to various treatments exposed for 5 h, at which time, migrating cells were quantified. Data shown are representative of three‐independent experiments with similar results. Bar graphs depict average ± SD for positive cells, ***p ≤ .001 (Student's *t* test).

To evaluate the effect of loss of Ca^2+^ signalling and understand if this is the mechanism leading to immune cell infiltration, we initially looked if ER stress could induce immune infiltration as observed in salivary glands. We seeded salivary gland cells on the bottom of the dish and placed immune cells (primary macrophages) in an insert to evaluate their migration upon various stimulations (Figure 5A). Salivary gland cells were either treated with DMSO (control), or with known ER stress/cell death inducers brefeldin A (BFA) or tunicamycin (Tuni) for 12 h, and immune cell migration was evaluated in each condition. Microscopic images and the quantification of the migration assay displayed an increase in the number of migratory primary macrophage cells under ER stress conditions (Figure 5A,B). Importantly, blocking Ca^2+^ entry (using SKF) also increased the number of macrophage cells migrated through the membrane (Figure 5A,B). Together, these results show that loss of TRPC1 function/expression that induces cell death (Figure S4G) could promote immune cell infiltration. To further establish that indeed this is the mechanism for immune cell migration, we took the conditional media from these cells (control, +BFA, +Tuni, +SKF) and placed them in the lower compartment (without salivary gland cells), along with immune cells in the insert and evaluated migration of macrophage cells. Interestingly, the primed media that was devoid of salivary cells was enough to induce the migration of primary macrophages (data not shown), suggesting that loss of acinar cells could release soluble factors that induce immune cell migration.

Data presented thus far support that loss of Ca^2+^ signalling‐induced cell death is an important factor that could contribute to immune cell migration thereby, contributing to pSS. To further identify the mechanism of how immune cell migration is induced, damage‐associated molecular patterns (DAMPs) that are released from dying cells were evaluated. DAMPs are sensed by innate immune cells,^28^ which in turn promotes immune cell migration and leads to the development of numerous inflammatory diseases.^40^, ^41^, ^42^ Interestingly, the level of DAMPs (histone‐3, HMGB1 and HSP70) released was significantly increased in the media in the presence of stressors or upon blocking TRPC1 function (Figure 5C). Furthermore, pro‐inflammatory cytokines were also significantly higher in the media of cells that were treated with BFA, Tuni or SKF‐96365 for 12 h (Figure 5D), which could be from migrated immune cells. Overall, these results show that induction of ER stress/cell death, which is due to loss of Ca^2+^ signalling (probably through TRPC1 that decreases saliva secretion), induces the release of DAMPs from salivary tissues that facilitates the migration of immune cells into salivary glands; however, the presence of ER stress in pSS is not identified. Thus, we next evaluated if ER stress/cell death is observed in pSS using salivary gland sections gender‐matched control and pSS samples. Importantly, expression of CHOP, which is a well‐known marker for ER stress, was increased in salivary tissues obtained from pSS patients (Figure 5E). In addition, CHOP expression was observed in both acinar and ductal cells suggesting that loss of Ca^2+^ signalling could be the trigger and thus might contribute to the disease. Consistent with these results, an increase in acinar cell death was also observed in salivary tissues obtained from pSS patients (Figure 5E), suggesting that indeed ER stress is present in pSS. Interestingly, 6‐month‐old IL14α Tg mice also showed an increase in HMGB1 expression when compared with age‐matched control mice (Figure 5F), suggesting that the release of alarmins could be the main reason for immune activation observed in salivary gland dysfunction. To further substantiate our results, we used alarmin inhibitor paquinimod and evaluated immune cell migration. Interestingly, cells pre‐treated with paquinimod showed inhibition in immune cell migration along with a decrease in alarmins (after 18 h) even in the presence of stressors or Ca^2+^ channel blockers (Figure 5G and S5 A–I), suggesting that the release of alarmins is the key event involved in immune activation and migration.

### 2.6 Paquinimod treatment reverts pSS phenotype in IL14α Tg mice

**FIGURE 6:**
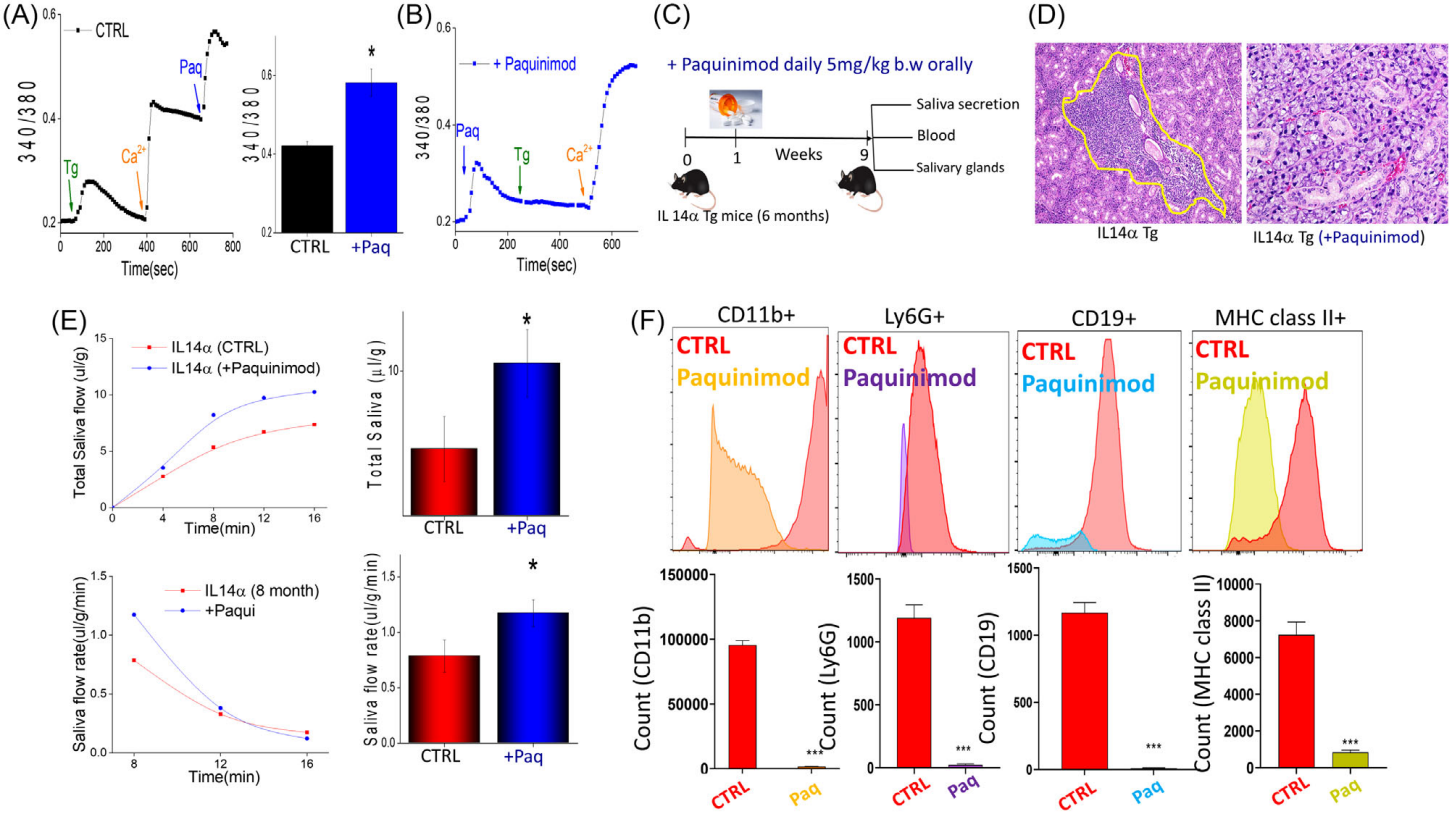
Paquinimod reduces infiltration of inflammatory cells and restore salivary gland function in IL14α transgenic mice. (A) Representative plots of Ca^2+^ imaging performed on Fura‐2AM loaded human submandibular gland (HSG) cells with bath application of paquinimod (300 µm) or pre‐treated with paquinimod (300 µm for 30 min) as indicated in part (B). Bar graphs show quantification of Ca^2+^ entry under control and paquinimod‐treated conditions from 120 to 140 cells. (C) Six‐month‐old IL14αTg mice were treated with paquinimod (*n* = 8) or saline (control, *n* = 8) for 9 weeks. Representative hematoxylin and eosin (H&E)‐stained salivary gland sections (*n* = 3) from saline‐treated IL14α Tg mice (control), or IL14α Tg mice treated with paquinimod for 9 weeks, showed few to no infiltration of immune cells. Scale bars shown are 100 µm (D) Total saliva secretion (*n* = 8) and rate of saliva in saline or paquinimod‐treated (for 9 weeks) IL14α Tg mice. (E) Quantifications of total saliva and saliva flow rate in each condition are shown as bar graph (average ± SD, *p ≤ .05 (Student's *t* test). (F) Single cell suspensions from salivary glands analysed by flow cytometry, and cells were stained with monocytes marker (CD11b+ AF594), dendritic cells marker (MHC class II+ AF488), B‐cell marker (CD19+ allophycocyanin [APC]) and neutrophils marker (Ly6G+ eFluor 450), showing a significant decrease in immune infiltrated in paquinimod‐treated IL14αTg mice. Data shown are representative of three‐independent experiments with similar results. Bar graphs (shown below) depict average ± SD for relative values, ***p ≤ .001 (Student's *t* test).

**FIGURE 7:**
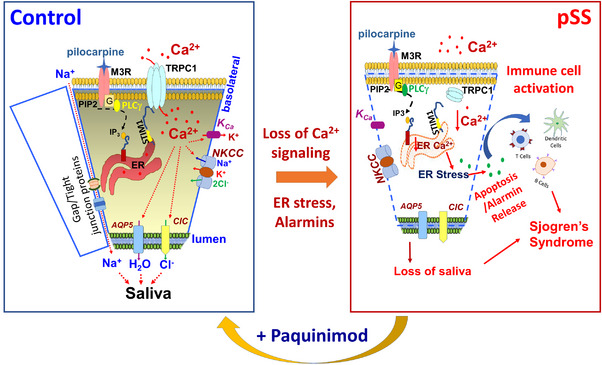
Schematic showing that loss of transient receptor potential canonical‐1 (TRPC1)‐mediated Ca^2+^ entry induces endoplasmic reticulum (ER) stress. Loss of Ca^2+^ signalling induces the release of alarmins needed for immune cell infiltration in salivary tissues. Moreover, paquinimod treatment restores Ca^2+^ entry, prevents alarmin release and reverts the development of primary Sjogren's syndrome (pSS) phenotype.

Data presented thus far establish that loss of Ca^2+^ entry leads to cell death that induces alarmin release that induces immune cell activation. Importantly, isolated acinar cells obtained from paquinimod‐treated IL14α Tg mice also showed an increase in Ca^2+^ entry, suggesting that it regulates Ca^2+^ signalling (Figure 6A,B), which can prevent alarmin release thereby inhibiting immune cells infiltration as observed before. We next evaluated if inhibition of alarmin release could prevent the development of pSS phenotype in IL14α Tg mice. Therefore, we designed experiments where we used paquinimod to suppress the effects of alarmins and see if it can decrease the development of the pSS phenotype. We used 6‐month‐old IL14α Tg mice and treated them daily (orally 5 mg/kg b.w) with paquinimod as shown in the schematic (Figure 6C). Excitingly, the 9 weeks of treatment of paquinimod successfully decreased immune cell infiltration in mouse salivary glands and histopathological analysis showed a decrease in immune foci, which was contrasted in non‐treated IL14α Tg mice (Figure 6C and Figure S5J). Subsequently, paquinimod‐treated mice showed an increase in both the total and the rate of saliva secretion in IL14α Tg mice (Figure 6D,E), suggesting that paquinimod treatment can restore salivary gland function. Finally, we analysed the presence of immune cells by flow cytometry and as expected, in comparison with the untreated group, paquinimod‐treated mice showed a decrease in CD11b^+^, Ly6G^high^, CD19^+^ and MHC class II immune cells (Figure 6F). We also showed the same trend in peripheral blood mononuclear cells (PBMCs) of treated and untreated mice, where there is a significant decrease in immune cells markers in CD11b+ (monocyte), Ly6G^high^ (neutrophils), CD19+ (B cells) and MHC class II (dendritic cells, B cells and monocytes/macrophages) (Figure S5 K–N). Importantly, an increase in Ca^2+^ entry was also observed in acinar cells isolated from submandibular glands of paquinimod‐treated IL14α Tg mice (Figure S5O). Finally, a decrease in alarmin release was observed in mice that were treated with paquinimod, when compared with vehicle‐treated IL14α Tg mice (Figure S5P). Collectively, these results show that restoration of Ca^2+^ signalling and/or inhibition of alarmins release by paquinimod decreases the migration of immune cells into salivary glands and restores salivary gland function, thereby reversing the pSS phenotype in IL14α Tg mice (Figure 7).

## 3 DISCUSSION

pSS is a complex disorder that leads to dry mouth/eyes, along with other systemic issues. Although loss of basal and stimulated saliva secretion is commonly observed in pSS, the molecular mechanisms that underlie the pathology are not established. Lymphocytic infiltration in the salivary glands as well as presence of autoantibodies in the blood are common features observed in pSS. Moreover, if immune cell infiltrations are the cause for the disease pathology or are needed to remove, the damaged/nonfunctional acinar cells are not known. In addition, the mechanism that leads to a decrease in saliva secretion in pSS remains poorly defined. In the present study, we show that both in a mouse model of pSS (IL14α Tg mice) and in human pSS patients, a decrease in saliva secretion, increased immune cell infiltration, and loss of TRPC1 function/expression was observed. Importantly, loss of salivary gland function was gradually decreased, and IL14α Tg mice older than 6 months of age showed significant saliva loss. In contrast, younger mice were resistant to the loss of salivary gland function, suggesting that age‐related changes could be critical for the disease progression. To identify the factors that could explain as why a decrease in saliva secretion was observed in aged IL14α Tg mice, we used a genomic approach that observed that a significant decrease in ion transport especially genes involved in Ca^2+^ signalling mechanism was decreased early on in IL14α Tg mice. Salivary gland function is regulated by the activation of muscarinic receptors which increase cytosolic Ca^2+^ levels in acinar cells.^21^, ^43^ Moreover, increase in [Ca^2+^]*_i_* in salivary gland acinar cells is critical for sustained saliva secretion as it regulates major ion channels, mainly the NKCC1, Ca^2+^ activated chloride/potassium/water channels that are required for saliva secretion.

Although two distinct Ca^2+^ entry channels Orai1 and TRPC1 have been identified,^21^, ^43^ previous studies from our lab shown that TRPC1 is essential for saliva secretion.^43^ Importantly, TRPC1 expression was less in pSS patients, and TRPC1 localization in the basolateral membrane was also decreased in pSS patients, which could explain the decrease in agonist mediated Ca^2+^ entry critical for saliva secretion. Consistent with the human data, expression of TRPC1 was also decreased in an age‐dependent manner, where the loss of TRPC1 was observed after 5 months of age, whereas no change in other TRPC or Orai1 channels was observed. Consistent with these results, a significant decrease in TRPC1 function was observed in 5‐month‐old IL14α Tg mice that not only inhibited Ca^2+^ entry but also decreased ER Ca^2+^ levels, which, together, is essential for protein synthesis and normal salivary gland physiology. Moreover, disturbances in ER and/or cytosolic Ca^2+^ homeostasis are linked with many diseases,^44^ but, its role in pSS is not clear. Importantly, 6‐month‐old IL14α Tg mice only showed minor presence of lymphocytic infiltrations in salivary tissues, whereas increased cell death was observed in older mice, which suggest that perhaps loss of Ca^2+^ signalling precedes immune cell infiltrations in salivary glands cells. Consistent with this data, TRPC1 KO mice showed increased immune infiltration in salivary gland cells, suggesting that loss of Ca^2+^ signalling may be the critical factor for salivary gland dysfunction.

To understand as how loss of TRPC1 function leads to immune infiltration, we focused our attention on ER stress and salivary gland cell death. Loss of ER Ca^2+^ homeostasis, which is due to the loss of Ca^2+^ entry, has been shown to induce the UPR. UPR is present in most cells that decreases abnormal protein synthesis to promote cell survival^45^, ^46^; however, if not controlled, it leads to ER stress, cell death and to the development of autoantigens, which are observed in all autoimmune diseases.^3^, ^47^ Our data suggest that inhibition of Ca^2+^ entry in salivary gland cells is the critical factor that lead to ER stress. Importantly, the presence of muscarinic 3 receptor autoantibodies in pSS patients, suggests that loss of saliva secretion in pSS could again be due to the loss of Ca^2+^ homeostasis. Moreover, alterations in the expression of UPR‐related molecules have been suggested to contribute to the initiation/development of autoimmune diseases; however, the mechanism is not known. Here, we show that loss of TRPC1 function is the main factor that leads to a decrease in ER Ca^2+^ levels and the initiation of ER stress. Importantly, the addition of ER stressors or inhibition of TRPC1 function showed an increase in immune cell infiltration into salivary gland cells. Interestingly, several immune cells, including dendritic and macrophage cells, are important for limiting infections and/or epithelial cell damage,^48^ and when damage is observed, they are activated by the release of alarmins. Loss of TRPC1 function led to increased cell death in acinar cells, as 6‐month‐old IL14α Tg mice also showed an increase in tunnel staining, which is a marker for cell death. Importantly, loss of Ca^2+^ signalling precedes salivary gland cell death and the infiltration of immune cells. Moreover, within 5 months, TRPC1 expression and its channel activity were decreased, suggesting that loss of TRPC1‐mediated Ca^2+^ entry must be one of the precipitating factors that could lead to the development of pSS. However, the precise mechanism as to why Ca^2+^ signalling especially TRPC1 expression was decreased in pSS remains to be evaluated. Importantly, Orai1 has been shown to modulate TRPC1 plasma membrane expression, which could be the mechanism for the decrease in TRPC1 expression. Nonetheless, our data provide a mechanism that shows that loss of TRPC1‐mediated Ca^2+^ signalling leads to ER stress that promotes immune cell migration/activation into salivary glands.

Another factor that has been linked to inflammation is the release of alarmins.^49^, ^50^, ^51^ Activation of the inflammasome is shown to respond to alarmin release in pSS.^52^ Our data provide the mechanism that a decrease in Ca^2+^ signalling is critical for the release of alarmins that could lead to immune cell activation/infiltration. In addition, IL14α Tg mice‐treated with paquinimod, which is known to inhibit alarmin release, also increased Ca^2+^ entry and was able to revert the pSS phenotype by decreasing immune cell infiltration and restoring salivary gland function. Interestingly, activated dendritic and other immune (T and B) cells have been shown to infiltrate into salivary glands in response to alarmins/chemokines,^53^ further suggesting that loss of Ca^2+^ signalling is the key event in immune cell activation/migration.^54^, ^55^ Although data from human studies are limited, the sequence of events identified using IL14α Tg mice could be promising to understand the mechanism(s) that leads to the pathogenesis of pSS. Interestingly, miR‐142‐3p is shown to be a pathogenic driver of immunopathology in pSS, which also inhibits Ca^2+^ signalling.^56^, ^57^ Similarly, agonist‐regulated intracellular Ca^2+^ release and Ca^2+^ entry are defective in acini from SS patients,^58^ which further supports our data and puts forward a new mechanism that could lead to pSS.

Our data show that in the presence of ER stress inducers, several key inflammatory cytokines: TNFα, IFNγ and IL1β/6 were increased, which could assist in the formation of lymphoid organ (T‐ and B‐cells accumulation) along with contributing to the loss of salivary cells. TNFα initiates apoptosis in salivary gland cells, and cell death is observed upon the loss of TRPC1 function/expression. Although the extent of apoptosis in acinar cells is observed, its relation to the development of pSS needs more research. Nonetheless, the data presented here clearly show that loss of Ca^2+^ signalling is one of the precipitating factors in all these events. Importantly, the abnormalities identified in pSS do not correspond to the presence of immune cells present in the gland; suggesting that the drastic decrease in salivary secretion observed in pSS is not primarily due to the presence of immune cells. Data presented here identify the mechanism, which shows that loss of Ca^2+^ signalling per se may induce ER stress that promotes acinar cell death and releases alarmins/cytokines/chemokines necessary for immune cell migration into salivary glands. Our study further showed the importance of restoring Ca^2+^ signalling and/or inhibition of alarmins in the development of pSS. Paquinimod treatment not only prevented alarmin release^3^, ^28^ but also restored Ca^2+^ signalling that improved salivary gland function along with decreased immune cell infiltration (Figure 7). These in vivo results are promising but clinical trials are needed to fully evaluate if paquinimod could benefit pSS patients. Nonetheless, the data strongly suggest that restoration of Ca^2+^ signalling and/or alarmin release could be of potential benefit to these patients.

## 4 METHODS

### 4.1 Animals and acinar cell isolation

Control (WT mice (C57BL/6) 1–17 months), TRPC1^−/−^ (months) and IL14α Tg mice (1–17 months of age) were used for these experiments. The animal protocol was approved by the institutional IACUC, and animals were randomized, blinded for the evaluator before the use in individual experiments. The phenotype and development of IL14α Tg mice and TRPC1^−/−^ used in here are previously characterized in Refs. [25, 32, 33]. Moreover, as pSS is mainly observed in women, only female mice were used. Animals were housed in a clean, sterile environment that had access to food and water and cared based on institutional guidelines. Mouse submandibular glands were used for the isolation of primary acinar cells. Briefly, mice were sacrificed, and individual salivary glands were isolated, minced and digested with collagenase‐II (2.5 mg/8 mL for 15–30 min at 37°C) in standard extracellular solution (SES) that was supplemented with .1% bovine serum albumin (BSA). Isolated salivary cells obtained were visible confirmed for single cell preparation washed twice with the SES buffer and re‐suspended in buffer prior to the use for the experiments. To investigate the effect of Paquinimod (Sigma, St. Louis, MO, USA) on IL14α Tg mice, mice (*n* = 10) were orally given using Polypropylene Feeding Tubes for Rodents (Instech, Plymouth Meeting, PA, USA). The compound was given daily at the concentration corresponding to daily doses of 5 mg/kg body weight/day. The treatment was continued for 9 weeks; afterwards, saliva secretion was evaluated and mice were sacrificed, and salivary glands as well as blood samples were collected for individual experiments.

### 4.2 HSG culture and silencing

HSG cells obtained from Dr. Ambudkar and were cultured in standard MEM medium that contained 10% FBS, penicillin (50 U/mL) and streptomycin (50 µg/mL). Cells were grown in tissue culture incubator till they reach 90% confluency and were passaged (using trypsin) as described in Refs. [23, 24]. For silencing experiments, 60% confluent cells were transfected with a pool of TRPC1siRNA duplexes (Dharmacon), using Lipofectamine‐RNAiMAX (Invitrogen, Carlsbad, CA, USA) in Opti‐MEM medium (no FBS). In addition, a non‐targeting NT‐siRNA (https://www.ncbi.nlm.nih.gov/gene?term = 7220) (Qiagen, Valencia, CA, USA) was used for control experiments, and cells were typically used for individual experiments 48 h post‐transfection.

### 4.3 Human samples

Human salivary gland samples were obtained from the University Medical Center Groningen (UMCG), where individual patients were evaluated for their assessment of pSS. The collection of samples was approved by the institutional Medical Research Ethics Committee at UMCG, and written informed consent was obtained from all patients. Individual patients were evaluated based on the 2016 ACR–EULAR criteria, and only patients who fulfil four out of six pSS classification criteria were identified as pSS patients. Patients who do not reach this threshold were used as controls (as shown in Figure S1A). Finally, tissue biopsies for both minor salivary and parotid glands were obtained, fixed, sectioned and used for staining as described later.

### 4.4 Saliva collection

Animals were randomly assigned to various groups as needed and anesthetized prior to the evaluation of the neurotransmitter‐induced saliva secretion. Individual mice were placed head down on warm cotton blanket to maintain the body temperature, injected with .5 mg/mL pilocarpine at 1 µL/g body weight, and saliva secretion was assessed. Pre‐weighed centrifuge tubes were used, and total saliva was collected at every 4 min intervals for a total length of 16–20 min. Following saliva collection, individual tubes were weighed again, and both rate and the flow of saliva were quantified as described previously in Refs. [25, 37].

### 4.5 Immunohistochemistry and confocal imaging

Human and mice salivary glands were obtained from individual experiments as described earlier. The tissues were washed twice in .1 M PBS buffer + 5% sucrose (pH 7.2), followed by the addition of 4% paraformaldehyde in the same buffer for 30 min. Following fixation, tissues from individual glands were placed at 4°C (overnight) in PBS that was supplemented with 20% sucrose. Individual samples were finally embedded in plastic dishes using Tissue‐Tek OCT compound and placed immediately on dry ice, and frozen tissues were used for obtaining individual sections (10 µm thick cryosections) using a cryotome (−20°C). For immune cells infiltration, H&E (Sigma, St. Louis, MO, USA) staining was used on individual sections. Following the staining, images from individual sections (minimum of 3 for each condition) were obtained using a light microscope (at 10 or 40× magnifications). To characterize the focus score in human samples, presence of immune cells was evaluated on the basis of the total number of foci (H&E staining) in each specimen. For evaluation of fluorescent staining, tissue sections from various animal groups or human salivary gland biopsies samples were used. Tissue sections obtained were permeabilized using .1% TritonX‐100 in PBS (pH 7.4) for 30 min, washed twice with PBS and blocked using donkey serum as described in Refs. [25, 37]. Samples were the incubated with individual primary antibodies and allowed to stay overnight (4°C) in a hydrated chamber. Individual slides were washed three‐times using PBS with 5% BSA (pH 7.4) and incubated with secondary antibodies that are conjugated with red/green fluorophores. Following the staining protocol, each slide was washed three‐times (PBS with 5% BSA) and mounted using VECTASHIELD (Vector Laboratories, CA, USA). For tunnel staining, tissue sections were stained using the tunnel assay kits (Abcam) and following the manufacturers protocol. Individual images were acquired at 40× or 63× magnifications using an LSM 510 Meta microscope (Carl Zeiss, Thornwood, NY, USA). Individual images were analysed using ImageJ software (NIH). For quantification, selected areas were confirmed at a high magnification that the infiltration of immune cells present was counted using the Hybrid cell counter.

### 4.6 [CA^2+^]*_i_* measurement

Individual mice were used, and salivary glands were isolated for obtaining single cell preparations as described earlier. Following collagenase digestion, digested cells were washed twice with SES buffer, looked under the microscope to confirm single acini/ductal cells. Suspended cells of 100 uL were placed on 35 mm glass‐bottomed culture dishes (MatTek, Ashland, MA, USA) along with 2 µM Fura‐2AM (Abcam) for 45–60 min at 37°C in a tissue incubator. To evaluate intracellular Ca^2+^, the culture dishes were gently washed with Ca^2+^‐free SES buffer and placed under the microscope for calcium measurement. Changes in Fura‐2AM fluorescence in single cells were measured by the addition of thapsigargin (1 µM) in a Ca^2+^‐free SES using a TILL Photonics spectrofluorometer (TILL Photonics In., Eugene, OR, USA). To measure calcium entry, 1 mM Ca^2+^ was added, and images were acquired every second as described previously.^25^, ^37^ Ratio of 340:360 was used to measure chances in cytosolic calcium levels and quantified.

### 4.7 Electrophysiology

For patch clamp experiments, cells placed on glass coverslips were transferred to the recording chamber, and individual acinar cells were identified visually. The outside bath solution (Ringer's solution) was continuously perfused which contains: NaCl, 145 mM; KCl, 5 mM; MgCl_2_, 1 mM; CaCl_2_, 1 mM; Hepes, 10 mM; glucose, 10 mM and adjusted to pH 7.0. Whole‐cell currents were initiated by the addition of thapsigargin, and currents were recorded using an Axopatch 200B (Axon Instruments, Inc.). The patch pipette used for measuring the current had resistances between 3 and 5 M after filling with SES solution containing: cesium methane sulfonate, 150 mM; NaCl, 8 mM; Hepes, 10 mM; EGTA, 10 mM, adjusted to pH 7.4 (CsOH). The osmolarity of the solutions used was between 305 and 315 mosm. Basal leak currents were subtracted from the final currents, and average currents are shown in the figures. The maximum peak currents were calculated at a holding potential of −80 mV, and the *I*–*V* curves from individual cells were made using a ramp protocol ranging from −100 to +100 mV and 100 ms duration was delivered at 2 s intervals and plotted.

### 4.8 Immune cell migration assays

Migration of immune cells was performed using 24‐well transwell plates that were placed with 8 µm pore inserts. HSG cells were placed overnight in the bottom of the dish and treated with 10 µM of tunicamycin (Tuni), 10 µM of BFA and 10 µM of SKF96365 for 12 h following previous protocol as described in Ref. [^59^]. Murine primary macrophage was resuspended in 100 µL of DMEM complete media and placed in the inserts. After 6 h, the inserts were removed, washed three times with PBS, and the numbers of migrated immune cells were fixed, stained with Trypan blue. Cell membranes were removed and mounted on glass slides for evaluation. Immune cell migration was identified and quantified using a standard light microscope as described in Ref. [^59^].

### 4.9 Flow cytometry

Mice were randomized for individual experiments, sacrificed and submandibular glands, and whole blood was collected. Glands were minced, and cells were separated using collagenase solution (Roche Diagnostics, Mannheim, Germany) in PBS (Ca^2+^ and Mg^2+^ free) for 1 h at 37°C, until leukocytes were released from the tissue. After digestion, the cell suspension was filtered through a 70‐µm nylon Falcon Cell Strainer (Becton Dickinson and Co.), and filtered cells were washed twice with PBS. Leukocytes obtained from submaxillary glands were resuspended in FACS buffer (1% BSA and .05% sodium azide in PBS) at a concentration of 2 × 10^6^ cells/mL. PBMCs were isolated by density gradient centrifugation using Ficoll‐Paque solution from whole blood of treated and untreated mice, and cells were washed with PBS twice before flow cytometer protocol was initiated. Surface staining was done using various combinations of antibodies labelled with allophycocyanin (APC), phycoerythrin (PE) and PerCP‐Cyanine5.5, Alexa Fluor 594 (AF594), eFluor 450. Fluorescent‐labelled anti‐mouse antibodies anti‐CD4 (PE), anti‐CD19 (PeCP‐Cy5.5), anti‐CXCR4 (APC), anti‐Ly6G (APC), anti‐CD11b+ (AF594), anti‐MHC class II (AF488) all labelled antibodies were used at 1:100 dilution. For flow cytometry, we acquired 10000–20000 events per sample. Data were collected on a FACS LSRII flow cytometer and analysed using FlowJo (San Jose, CA, USA).

### 4.10 RNA sequencing and RT‐PCR

Salivary tissues from individual animals were obtained as described earlier, and total RNA was isolated using Trizol reagent as per manufacturers protocol. Ribosomal RNA was depleted using standard protocols, and RNA obtain was quantified (RIN values), before the construction of sequencing library (NEB Next Ultra RNA Library Prep Kit [New England Biolabs, Ipswitch, MA, USA]). The libraries obtained from individual samples were sequenced using the Illumina HiSeq instrument. The sequence data files (.bcl files) were converted into FASTQ files and de‐multiplexed using Illumina's bcl2fastq 2.17 software. Principal component analysis was used to establish correlation between individual samples using the DESeq2 R package. For quantitative PCR, RNA isolated from individual samples was quantified, and equal amount of total RNA was used. RT‐PCR was carried out using SYBR Green SensiMix (Quantace) using a Roche Light Cycler 480.

### 4.11 Measurement of alarmins and cytokines levels

HGMB1, histone‐3, hsp70, IL‐6, IL‐1β, IFNy, and TNFα levels were evaluated from the supernatant obtained from individual samples using the Enzyme‐Linked ImmunoSorbent Assay (ELISA) kit (Life Technologies, Carlsbad, CA, USA). All protocols used were standard that follows the manufacturer's instructions as shown in Refs. [53, 59].

### 4.12 Membrane preparations and Western blot analyses

Crude membranes from HSG cells or salivary tissue were isolated as described previously.^25^, ^27^, ^37^ Protein concentrations from individual samples were evaluated using Bradford reagents (Bio‐Rad), and 25 µg of total proteins were separated using a NuPAGE 4%–12% Bis–Tris gel (Invitrogen, Carlsbad, CA, USA). Proteins were transferred on to membranes, and Western blotting was done as shown in Refs. [53, 59] using individual antibodies as labelled.

### 4.13 Statistical analysis

Data obtained were analysed using Origin 9.0 (OriginLab, Northampton, MA, USA) and Graphpad prism 8.0 (San Diego, CA, USA). To establish significance, statistical comparisons were performed using one‐way analysis of variance on Ranks test followed by the Dunn post hoc for multiple comparisons or Student's *t* test as needed, and *p* values are indicated. Data obtained are expressed as means ± SD or means ± SE as labelled.

### 4.14 List of antibodies

**Table:** 

Antibody	Host	Clonality	Dilution factor	Company
CD45R/B220 (RA3‐6B2)	Rat	Monoclonal	1:50	Cell Signaling
CD3 (17A2)	Rat	Monoclonal	1:200	Cell Signaling
CD11c (D3V1E)	Rabbit	Monoclonal	1:200	Cell Signaling
CD19 (eBio1D3 (1D3))	Rabbit	Monoclonal	1:1000	ThermoFisher
CD4 (GK1.5)	Rabbit	Monoclonal	1:1000	ThermoFisher
CD194 (CCR4) (D8SEE)	Rabbit	Monoclonal	1:1000	ThermoFisher
CHOP	Rabbit	Monoclonal	1:1000	ThermoFisher
Anti‐TRPC1 antibody	Rabbit	Polyclonal	1:200	Alomone Labs
Anti‐TRPC5 antibody	Rabbit	Polyclonal	1:200	Alomone Labs
Anti‐TRPC6 (extracellular)	Rabbit	Polyclonal	1:200	Alomone Labs
ORAI1 (3F6H5)	Rabbit	Monoclonal	1:1000	ThermoFisher
STIM1 (D88E10)	Rabbit	Monoclonal	1:1000	Cell Signaling
β‐Actin (8H10D10)	Rabbit	Monoclonal	1:1000	Cell Signaling
CD11b (M1/70)	Rabbit	Monoclonal	1:1000	ThermoFisher
Ly‐6G (1A8‐Ly6g)	Rabbit	Monoclonal	1:1000	ThermoFisher
MHC class II (HIS19)	Rabbit	Monoclonal	1:1000	ThermoFisher

## CONFLICT OF INTEREST STATEMENT

The authors have declared that no conflict of interests exists.

## Supporting information

Supporting Information

Supporting Information

Supporting Information

Supporting Information

Supporting Information
