# Supplementary figures and images for: Targeting alarmin release reverses Sjogren's syndrome phenotype by revitalizing Ca2+ signalling
Source: Clin Transl Med. 2023 Apr 3;13(4):e1228. doi: 10.1002/ctm2.1228 (PMC10068318; doi:10.1002/ctm2.1228)

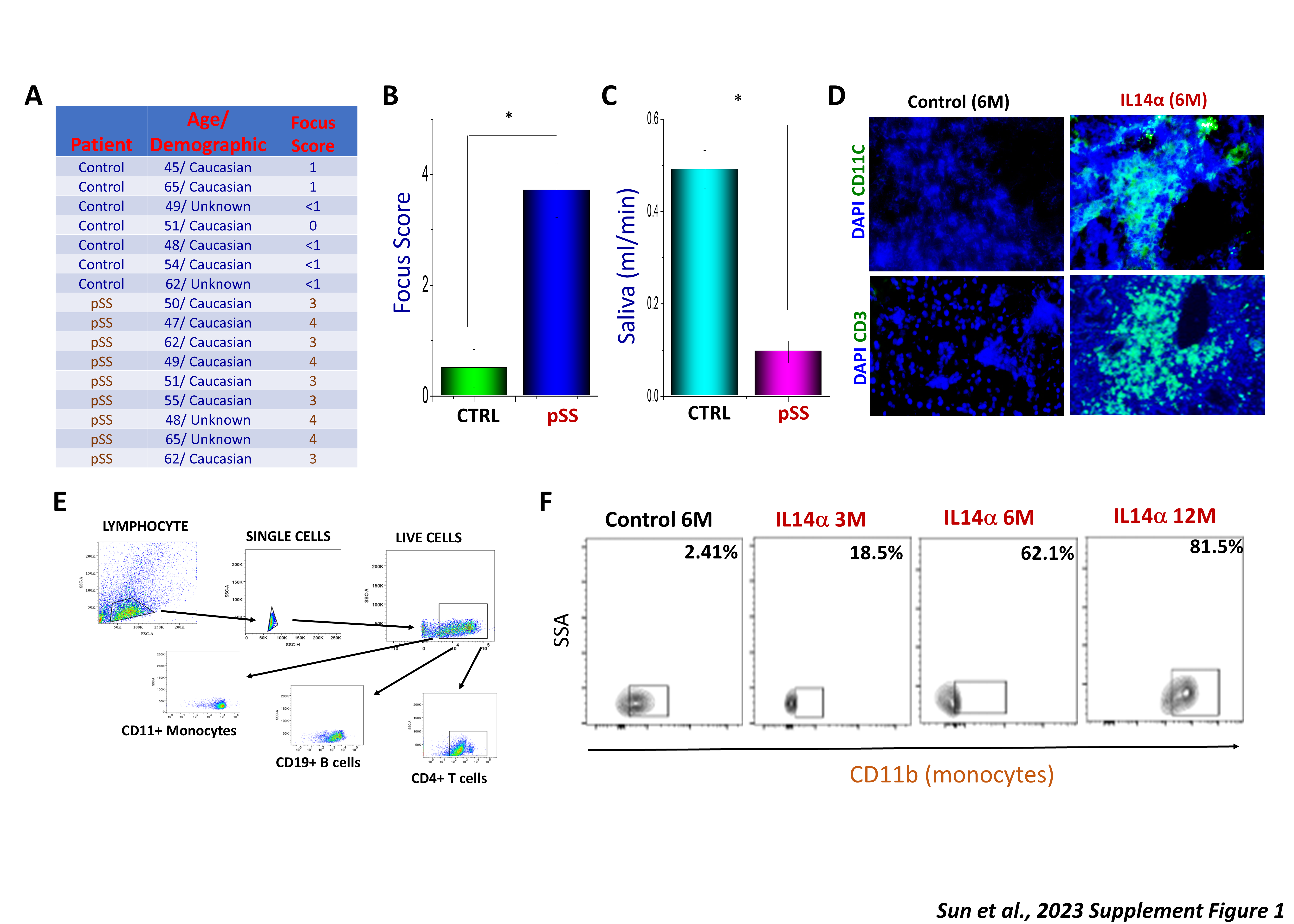

Supplement: Supplementary file 1 — Supporting Information [file CTM2-13-e1228-s001.TIF]

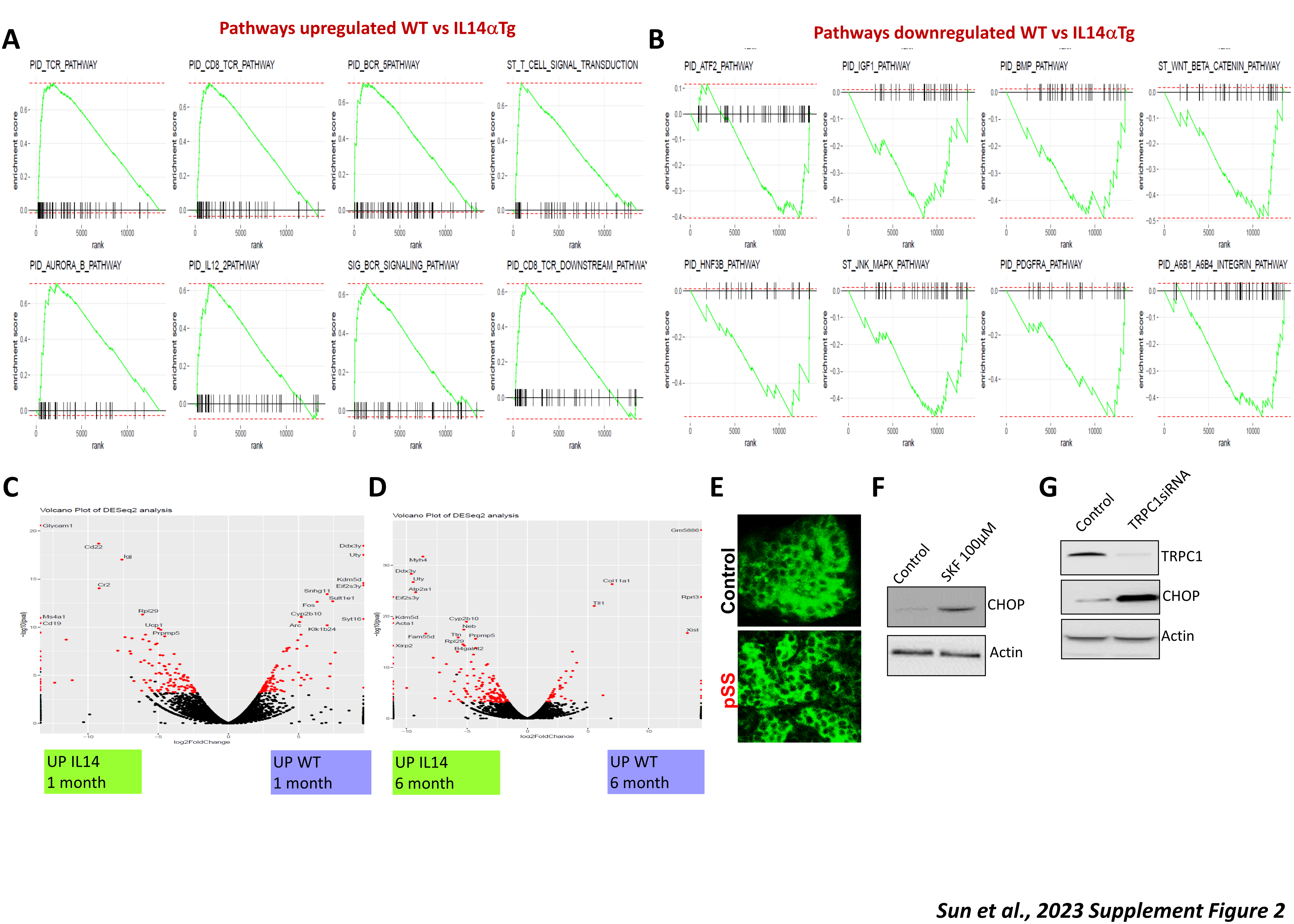

Supplement: Supplementary file 2 — Supporting Information [file CTM2-13-e1228-s003.TIF]

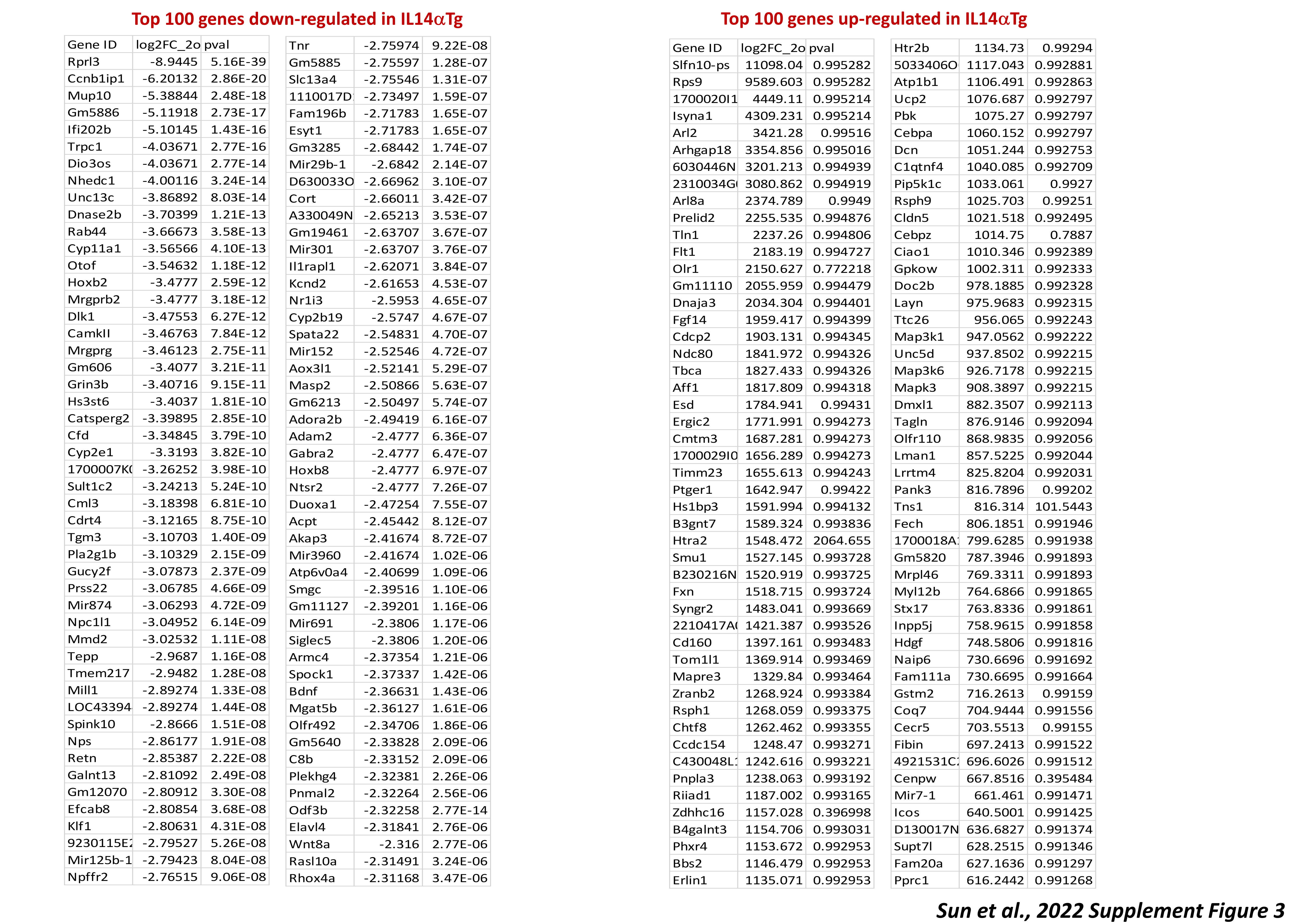

Supplement: Supplementary file 3 — Supporting Information [file CTM2-13-e1228-s004.TIF]

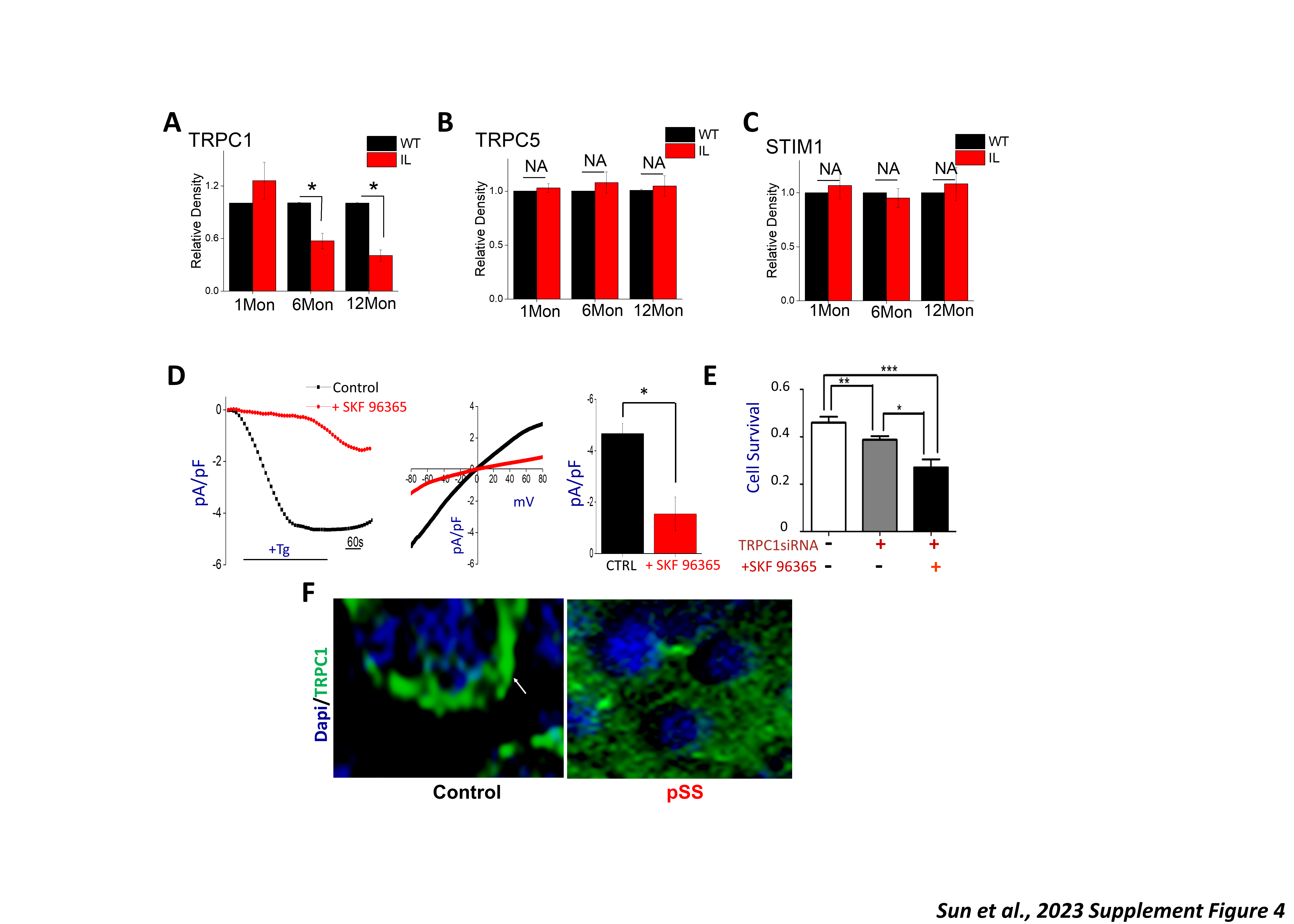

Supplement: Supplementary file 4 — Supporting Information [file CTM2-13-e1228-s002.TIF]

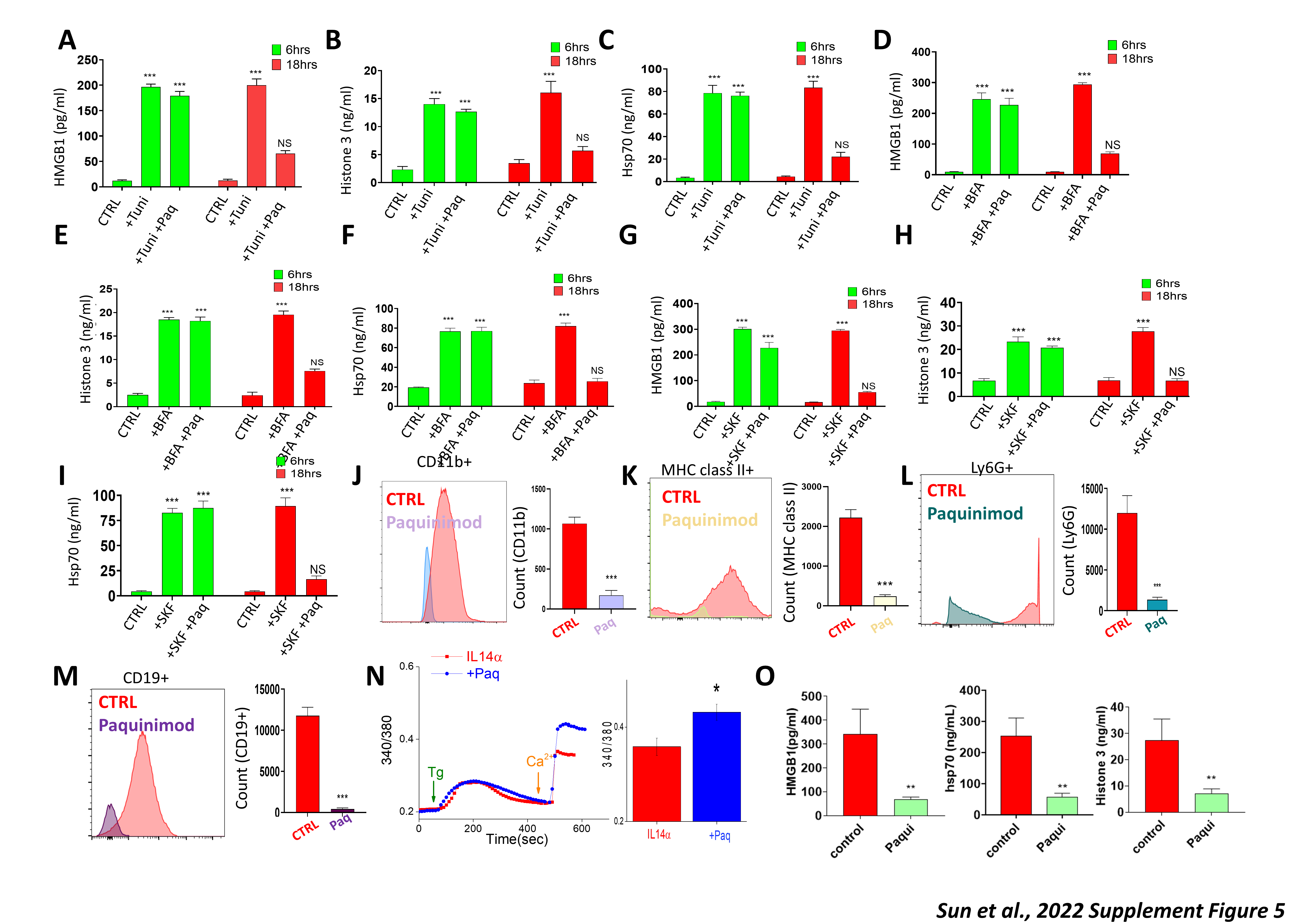

Supplement: Supplementary file 5 — Supporting Information [file CTM2-13-e1228-s005.TIF]
